# Gold Nanorods for Drug and Gene Delivery: An Overview of Recent Advancements

**DOI:** 10.3390/pharmaceutics14030664

**Published:** 2022-03-17

**Authors:** Atieh Jahangiri-Manesh, Marziyeh Mousazadeh, Shirinsadat Taji, Abbas Bahmani, Atefeh Zarepour, Ali Zarrabi, Esmaeel Sharifi, Mostafa Azimzadeh

**Affiliations:** 1 Department of Nanobiotechnology, Faculty of Biological Sciences, Tarbiat Modares University, Tehran P.O. Box 14115-154, Iran; a.jahangirimanesh@modares.ac.ir (A.J.-M.); m.mosazadeh@modares.ac.ir (M.M.); shirintaji@modares.ac.ir (S.T.); 2 Institute for Nanoscience & Nanotechnology (INST), Sharif University of Technology, Tehran P.O. Box 14588-89694, Iran; inmehr@gmail.com; 3 Department of Biomedical Engineering, Faculty of Engineering and Natural Sciences, Istinye University, Sariyer, Istanbul 34396, Turkey; atefeh.zarepour@gmail.com; 4 Department of Tissue Engineering and Biomaterials, School of Advanced Medical Sciences and Technologies, Hamadan University of Medical Sciences, Hamadan P.O. Box 65178-38736, Iran; 5 Medical Nanotechnology & Tissue Engineering Research Center, Yazd Reproductive Sciences Institute, Shahid Sadoughi University of Medical Sciences, Yazd P.O. Box 887-89165, Iran; 6 Department of Medical Biotechnology, School of Medicine, Shahid Sadoughi University of Medical Sciences, Yazd P.O. Box 887-89165, Iran

**Keywords:** gold nanorods, drug delivery, gene therapy, photothermal therapy, photodynamic therapy, theranostics

## Abstract

Over the past few decades, gold nanomaterials have shown great promise in the field of nanotechnology, especially in medical and biological applications. They have become the most used nanomaterials in those fields due to their several advantageous. However, rod-shaped gold nanoparticles, or gold nanorods (GNRs), have some more unique physical, optical, and chemical properties, making them proper candidates for biomedical applications including drug/gene delivery, photothermal/photodynamic therapy, and theranostics. Most of their therapeutic applications are based on their ability for tunable heat generation upon exposure to near-infrared (NIR) radiation, which is helpful in both NIR-responsive cargo delivery and photothermal/photodynamic therapies. In this review, a comprehensive insight into the properties, synthesis methods and toxicity of gold nanorods are overviewed first. For the main body of the review, the therapeutic applications of GNRs are provided in four main sections: (i) drug delivery, (ii) gene delivery, (iii) photothermal/photodynamic therapy, and (iv) theranostics applications. Finally, the challenges and future perspectives of their therapeutic application are discussed.

## 1. Introduction

The applications of nanotechnology in our lives are constantly increasing by way of innovations in design, synthesis, and functionalization of nanomaterials and nanostructures [1]. Those advancements have been applied in many fields to overcome past limitations and create new applications. The nano dimension brings new properties mainly because of the restriction in the electron motions of the particles and quantum confinement, which is also dependent on the material’s shape [2]. Some nanoscale-specific physiochemical properties include high surface area, outstanding electronic and optical properties such as localized surface plasmon resonance (LSPR) for noble metal nanoparticles, high mechanical strength, ease of functionalization, enhanced thermal conductivity and magnetic properties [3]. These traits make nanoparticles a great choice for various applications [3]. Nanoparticles can be applied in many different aspects including electrochemical applications (as electrolytes in batteries) [4,5], catalytic applications [5], solar cells [6], biosensors [7,8], imaging [9,10], therapeutic purposes [11,12], biomolecules tracking [13,14], tissue engineering [15,16] and gene or drug delivery [17,18].

As one of the most important applications of nanotechnology, nano-drug delivery systems (nDDSs) have attracted remarkable attention. nDDSs have unique features such as easier cell uptake, controlled drug release, prolonged stability of drugs inside the cells, long-lasting circulation time, reduction in drug side effects, enhanced bioavailability and biocompatibility, the potency of targeted delivery, lower administration dose [19,20], controllable pharmacokinetics, and traceability of delivery system [21,22]. These systems could be designed in the form of liposome [23], micelles, polymers [24], polysaccharides [25], self-assembled peptides [26], dendrimers, silica-based nanomaterials, bioactive glasses [27], hydrogels, carbon-based nanomaterials [28], metal nanoparticles, recently exosomes [29] and several other forms which could be used for delivering both drugs and oligonucleotides (DNAs or RNAs) [30,31,32,33,34,35,36]. Some examples of commercially available drugs containing nDDSs are Doxil^®^ (Doxorubicin, polyethylene glycol (PEG)ylated liposome) [19,37], Abraxane^®^ (Paclitaxel, Polymer nanoparticle) [38,39], and Vivagel^®^ (Dendrimer nanoparticle) [32,40]. There are also some nanoparticles used for delivery systems in clinical trials such as polymeric micelles (Paclitaxel, Phase II Trial) [41], dendrimers (Docetaxel, Phase II Trial) [42] and vaccination platforms for antigen delivery in cancer treatments [43].

nDDSs are considered systems in which therapeutic agents are introduced to the body with improved properties [44,45]. For example, *P*-glycoproteins which are expressed in cancer cells, pump antitumor drugs out of the cells. It has been evidenced that nDDSs can transfer anticancer drugs to the cell without being pumped out [22,46,47]. In addition, the blood–brain barrier (BBB), which does not allow drugs to pass through, can also be challenged by nDDSs [31,48]. Gulyaev et al. showed that doxorubicin with polysorbate 80-coated nanoparticles could be transported to BBB [49] via apo-lipoprotein E [50].

To understand the main mechanism in which nanomaterials can lead to good and efficient drug delivery, the interaction between nanomaterial and biological environment should be studied [22]. Therapeutic elements can be absorbed or attached to the surface or encapsulated/trapped inside the carrier. Covalent bonding, the strongest type of binding, is a strategy to control the administered dosage of the drug component [51]. Then, drugs can be released from nDDSs in response to the changes in the physiological environment such as temperature, pH, reducing nature, enzymatic activity or even concentration [52]. There are two possible mechanisms for enhancing drug availability at the targeted sites: (1) active delivery, in which some binding/recognition elements such as antibodies or aptamers are functionalized on the surface of carriers to develop a targeted DDS; (2) passive delivery, which only has the role of carrying drug particles through the reticuloendothelial system. Nano-drug delivery systems may be designed for both mechanisms [31,53]. The mechanism through which nanoparticles are passively delivered to the tumor site is called the enhanced permeability and retention (EPR) effect [54]. Due to the dysfunctionalities in vascular nature of solid tumors, the high molecular weight material such as nanoparticles will accumulate in the solid tumors and increase its permeability to drugs. This effect increases the nanodrugs entrance to the tumor sites [55]. This phenomenon, which was firstly discovered by Maeda in the mid-1980s, has different affecting factors such as the location and type of the tumor. For example, EPR effect in the brain tumors is very low due to the BBB presence [56]. Furthermore, high tumor interstitial fluid pressure (IFP), poor blood flow inside tumors, and abnormal vascular distribution compromise the EPR effect [57,58].

**Scheme 1:**
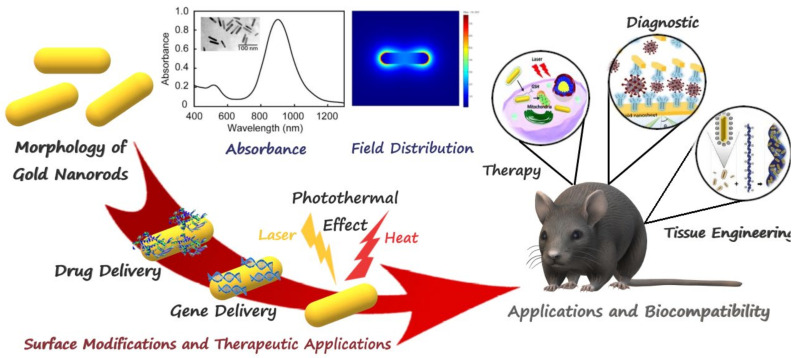

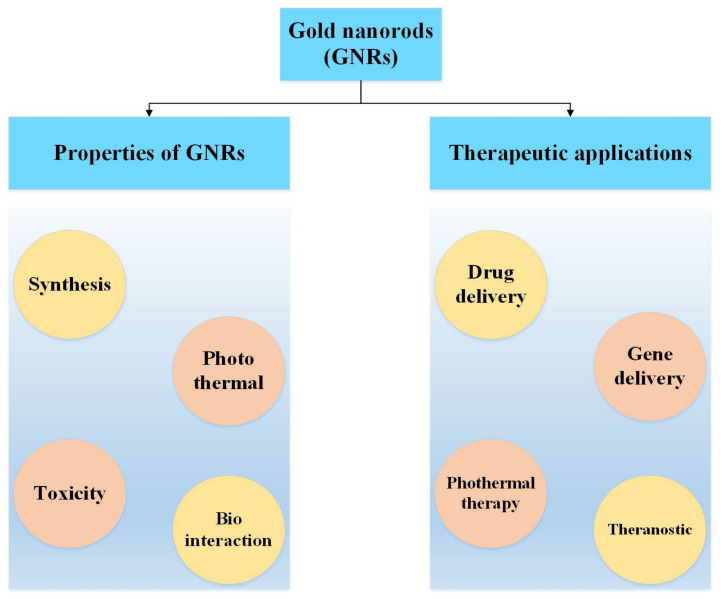
Overview of GNRs properties and medical applications.

Among different conventional used nanomaterials, gold nanostructures have attracted great interest, making them one of the most important platforms in nanobiotechnology and nanomedicine [59,60]. The significance of gold nanomaterials in medicine originates from their optical properties, which strongly depends on their size, aspect ratio and shape [61,62]. Due to the unique optical properties of gold nanomaterials, their ease of functionalization, and their high biocompatibility, gold nanomaterials have been increasingly used in medical applications, with potential use as carriers [62,63] for delivery of antigens for vaccination [64], gene delivery [65], other therapeutic targets [66,67]. Rod-shaped gold nanoparticles (gold nanorods or GNRs) have unique properties useful in medical applications. In this review, the synthesis methods and the properties of the GNRs are introduced and their therapeutic applications (drug/gene delivery, photothermal/photodynamic therapy, and theranostics) are focused and the challenges and future perspectives of their therapeutic application are discussed. Scheme 1 is representing general applications and properties of the GNRs discussed in this review.

## 2. Properties and Synthesis of GNRs

Gold nanorods are tiny rod shape structures with unique physical, optical and chemical features that make them favorable candidates in medical research [68]. The optical property of these tiny nanostructures returns to LSPR [69,70], which is an extraordinary optical property of metal nanoparticles induced by an incident electromagnetic wave in a particular wavelength; hence, it leads to collective oscillation of electrons [71,72]. Anisotropic structure of GNRs exhibit two surface plasmon bands in the UV–Vis spectrum, which are related to surface electron oscillation toward the transversal side of the particle and longitudinal side of GNRs [73]. Their ability to bind amine and thiol groups of other biomolecules facilitates their functionalization and bioconjugation processes and makes them ideal for use as nanocarrier systems [70,74].

### 2.1. GNR Synthesis Methods

One of the other advantages of GNRs is related to their accessibility and synthesizing of particles. Herein, there are quite number of synthesis methods which are divided into three group methods including chemical, physical and green synthesis method [62]. Despite of recent interests to replace more eco-friendly methods with less deposit problems and less toxicity, some limitations due to size controllability and synthesis yield led to those particularly chemical methods, are mostly preferred for GNRs synthesis among other types of chemical such as photochemical or electrochemical methods [62]. Electrochemical, seed-mediated, and synthesis with AgNO_3_ are the most common GNR synthesis methods. Electrochemical method has a high-quality yield; seed-mediated is the most favorable method with high yield, monodispersity, and ease of fabrication; AgNO_3_ method has up to 50% yield and is affected by many variable factors [75]. Unknown mechanism for some of the synthesis methods and their dependency on the environmental factors could negatively affect the reproducibility and controllable high yield production of GNRs [75] for industrial purposes as a main market limitation. The method called “seed-mediated growth” has been the most common method of producing colloidal GNRs (in 20–100 nm size range) since the 1920s [62]. To explain, GNRs are sequentially synthesized in a way in which Au salts are first reduced by reductants such as Citrate, forming spherical gold particles (seeds). Then, in a second step, adding reducing agents will induce assembling the gold spherical particles to produce gold nanorods as well [76]. Advances in synthesis could lead to the introduction of a new seed-mediated synthesis protocol that was proposed by Murphy et al. for the first time, by adding a kind of surfactant, so-called cetyltrimethylammonium bromide (CTAB), that clearly induces the zipping mechanism, aiming to achieve quite good rod morphology and better aspect ratio of GNRs. In this regard, the structural modification of GNRs is due to the fact that the CTAB works as a template for one-dimensional growth of these nanoparticles. Other surfactants will also affect the structural modifications [77]. Since then, different versions of optimizations have been published, such as the optimized protocol by Tohidi Moghadam et al. in Iran [78].

### 2.2. Photothermal Effect

GNRs have unique physicochemical properties, but undoubtedly, the most distinctive characteristics are due to their photothermal therapy effect, which can be defined as converting NIR radiation (650–900 nm) to heat energy [72]. Therefore, local heat generation leads to provoking cell death mainly via apoptosis and necrosis, even in deeper sides of soft tissues, especially for cancer therapy. Indeed, in recent years GNRs have been widely implemented in multiplexed cancer studies and could be useful in combating barriers of conventional cancer medications [72,79,80]. Recent studies have reported that some parameters such as aspect ratio of nanoparticles (length/width of a particle), radiation wavelength (Selected wavelength of NIR radiation raging 700–2000 nm), and coating (Covering agent of particle) can directly control the depth of penetration and efficacy of irradiation. For example, several experimental investigations have emphasized that 808 nm wavelength is an optimal point of laser radiation [81,82].

### 2.3. Toxicity Related to GNRs’ Surface Modifications

Nanoparticles such as GNRs are novel advanced materials, so their toxicity and safety issues should be under consideration by researchers. Accumulation, uptake, pharmacokinetics, and clearance are effective factors in toxicity analysis. Since these factors are different between nanomaterials and biomolecules, the biocompatibility and toxicity should be studied [83]. In terms of accumulation, nanoparticles are opsonized by resident macrophages in the tissues and then highly present in some healthy organs such as liver and spleen. This nonspecific presence and distribution may have toxicity and side effects on these healthy organs [84]. Uptake is basically the regulated cellular uptake (nanoparticles entrance to the cells) by mechanisms such as receptor-mediated endocytosis [85]. The pharmacokinetics feature represents the profile of nanoparticles’ bio distribution and clearance among time and the volume of utilized nanoparticles [86]. The final term, clearance, is the deletion of nanoparticles from the systematic circulating blood vessels [87]. Studies have shown that the surface modification of GNRs has the most impactful effect on their cytotoxicity. In research conducted by Alkilany et al., the toxicity of GNRs coated with polyelectrolytes (PE-GNRs) and polyethylene glycol (PEG-GNRs) in the therapeutic concentration of 1 nM were investigated on the vascular endothelial and smooth muscle cells, which were isolated from rat aortic rings. The results showed that PE-GNRs were toxic for vascular endothelial cells but not for the smooth muscle cells, while PEG coated nanorods did not have a toxic effect on vascular endothelial and smooth muscle cells. PE-GNRs cannot harm smooth muscle cells because the endothelium layer and sub-endothelial connective tissue are separated from direct contact with the GNRs. The uptake of PE-GNRs in vascular endothelium rather than PEG-GNRs is one the main reasons make it toxic for these cells. PE-GNRs induce significant changes in the vasorelaxation response which was measured by the tension changes in the aortic ring. Furthermore, the nitric oxide increase (as an inflammation indicator), and microscopic studied of the cells have shown toxic effects on vascular cells [88].

In the case of direct injection of GNRs into the blood vessels, their toxicity should be considered cautiously. Additionally, blood vessels are a route most nanoparticles come through during their transport in the body. Due to the small size of nanoparticles, their wide bio distribution in the body negatively affects the hemocompatibility in two ways: affecting the coagulations pathways and changing the blood cellular fates; disrupting the hemostatic balance, disseminated intravascular coagulopathy (DIC), and deep vein thrombosis (DVT) are hazardous effects of nanoparticles on hemocompatibility [89]. Mitochondria is one of the cell components which can be highly affected by the nanoparticles and changes the fate of the cells. In a study conducted by Nunes et al., the effect of CTAB-GNRs on mitochondrial integrity was evaluated. CTAB-GNRs decrease the respiratory ratio and phosphorylation pathways in the mitochondria and disrupt the electron chain transport. These results validate the toxicity of CTAB-GNRs by affecting the mitochondrial integrity [90].

On the other hand, the more a nanoparticle is used, the more environmental contamination it will have. So, in various research, the effect of GNRs on aquatic organisms was studied. Fish are the most used animal models in the environmental contamination investigations. In a study conducted by Souza et al., after the fish were subjected to sub-lethal exposure to the CTAB-GNRs (LC_50_ = 0.03 mg/L), the fish model showed a decrease in the neonate and an increase in the reactive oxygen species (ROS) level, which shows the increased toxicity levels in the fish [91]. In another similar study, conducted by Mesquita, after fish were exposed to CTAB-GNRs, no DNA damage was seen in the fish model, while malformation in the body development was observed even in the low doses (LC_50_ = 110.2 μg/L) [92].

To compare the toxicity effect of shape and coating, GNRs and gold nanoparticles (GNPs) with two different coatings, (polyethylene imine (PEI) and bovine serum albumin (BSA)), were used. Here, four different types of nanomaterial (PEI-GNRs, BSA-GNRs, PEI-GNPs, and BSA-GNPs) were synthesized, and their effects in different biological liquids such as serum, buffer, and cell culture media were investigated. It was showed that GNPs are less toxic and more biocompatible than GNRs. Since the surface functionalization of GNRs is critical in their cytotoxicity, this research proved that PEI-GNRs are nontoxic and safe and could easily penetrate normal and cancerous cells without damaging them [93].

### 2.4. The GNRs–Proteins Interaction

Since active biological material has a strong structure–function relationship, an appropriate carrier for drug delivery systems should also have no effect on the cargo’s structure. Since protein-based drugs could be one of the GNR’s cargos in nDDSs, investigating the effects of nanoparticle morphology on the protein’s structure is also important. GNRs and GNPs were both decorated with BSA, which showed that BSA’s biological activity was lost when it was joined with GNRs, while it did not change with the GNPs [94]. Thus, it seems that GNRs could unfold the protein cargo, while GNPs do not have such an effect, and therefore, GNPs could be better options for protein cargos.

## 3. GNRs for Therapeutic Applications

GNRs can be functionalized and simultaneously loaded by drugs or genes, acting as promising nanocarriers for targeted delivery of cargo. The load in such systems can be released in the designated location through the heat generation following NIR radiation. Thus, GNRs can be applied in enhancing the efficiency of targeted treatments. They are also considered as promising photothermal therapy agents. In this section, we have reviewed recent applications of GNRs for therapeutics purposes in three categories: drug delivery, gene delivery, and hyperthermia/photodynamic therapy.

### 3.1. GNRs for Drug Delivery Systems

**Figure 1:**
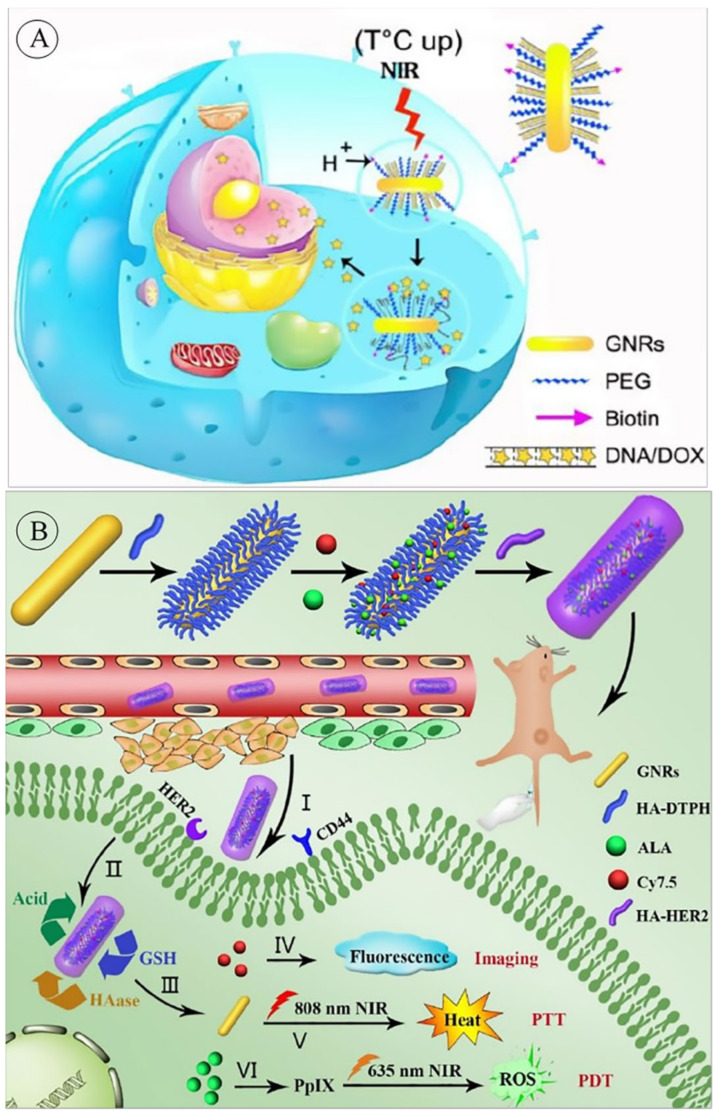
(**A**) Schematic drawing of the pH and NIR-responsive nDDS. Cancerous cells with overexpressed biotin receptors will uptake the Biotin-PEG-GNR-DNA/DOX system and enter its endosomes. Following the maturation of the endosomes, the pH will decrease, and thus DOX will be released. The system can alternatively be stimulated by NIR radiation for a local release. Reprinted from [95] with permission from Elsevier, Amsterdam, Netherlands. (**B**) The schematic representation of tri-sensitive nDDS for simultaneous photothermal and photodynamic therapy and fluorescence tracing for HER2/CD44 positive breast cancer cells. Reprinted from [97] with permission from Elsevier, Amsterdam, Netherlands.

There are quite many numbers of research investigating GNRs application in drug delivery systems for various purposes. Zhang et al. designed a nanocarrier for targeted drug delivery, responsive to pH and NIR, by conjugating a thiolated pH-responsive DNA to GNRs. A thiolated poly (ethylene glycol)-biotin was used to passivate the nanocarrier to provide a particular binding to cancer cells that showed over-expression of biotin receptors. Doxorubicin (DOX) was then intercalated in the double-stranded pH-responsive DNA (Figure 1A). Lower pH environment (pH~5) and/or 808 nm radiation resulted in the effective release of DOX. It was reported that the designed nanomedicine improved cell uptake remarkably and reduced drug efflux by the multidrug-resistant (MDR) breast cancer cell lines [95].

GNRs can also be used for improving skin permeability of proteins in the case of transdermal protein delivery, which can be an interesting method for therapeutic applications or for vaccination purposes. Haine et al. developed a method to enhance transdermal protein delivery by conjugating a polysaccharide-based hydrogel containing fluorescein isothiocyanate-modified ovalbumin (FITC-OVA) with GNRs on its surface. Using GNRs’ photothermal property generated heat due to a continuous-wave laser irradiation that led to an increase in the skin temperature and thus facilitated the protein’s permeation [96].

Using the GNRs’ photothermal effect, nanosystems can be produced for photothermal therapy or drug delivery systems. Niidome et al. designed an NIR-responsive system by modifying GNRs with poly (*N*-isopropyl acrylamide) gel. When the system was irradiated, the photothermal effect of GNRs led to a phase transition effect that converted the polymer layer to be hydrophobe, so the whole system shrank and accumulated in the irradiated tissue [98].

Innovative targeted and sustained drug releasing systems have also been integrated with GNRs relying on their electrical properties to achieve a natural heart-similar electrical conductivity. Since these particles could improve the conductivity of the hydrogels, Chen et al. designed an injectable bioactive hydrogel with high drug loading efficiency for myocardial infarction treatment [99]. They incorporated Astragaloside IV nanoparticles and GNRs as drug and conductivity enhancers, respectively, in a hydrogel formed from polyethylene glycol diacrylate/4-vinyl phenylboronic acid (PEGDA-PBA) and thiol hyaluronic acid (HA-SH). The system showed effectiveness in improvement of cardiac dysfunction and cardiac remodeling [99].

GNRs could also be coupled with fluorescent particles to design traceable drug delivery systems. These nanoparticles were used to design a system with photothermal and photodynamic therapeutic properties and traceability through fluorescent imaging. Photodynamic therapy is a two-step treatment in which a photosensitizer is activated by light stimulation, produces ROS that causes cell death. In their designed system, gold nanorods functionalized with hyaluronic acid, 5-aminolevulinic acid (ALA) and Cy 7.5 were used for photothermal treatment, photodynamic treatment, and fluorescent imaging. After entering the target cells, this system is destroyed in response to hyaluronidase, acidic pH and glutathione within the cell, and as a result of the separation of its components, the mentioned properties appear (Figure 1B) [97].

Herein, a comparative summary of the GNR-based nDDS is provided in Table 1.

An important property for materials to be used in drug delivery systems is non-(or low) toxicity. As described before, there are also efforts to reduce GNR’s toxicity and make them more appropriate for medical applications. PEG is widely used in this field, since it both improves GNRs’ stability and prevents the immuno-uptake of these nanomaterials [133]. In research by Niidome et al., mPEG-SH was added to surfactant-stabilized GNRs (hexadecyl trimethyl ammonium bromide or CTAB surfactant) to reduce its cytotoxicity. Intravenous injection of the PEG-GNRs to the mice showed that 54% of the injected material was found in the blood rather than accumulation in the liver, unlike the original CTAB-GNRs [134]. However, their method resulted in stability issues of some GNRs, which were later addressed by additional steps in the ligand exchange process, such as using Tris-HCl buffer [135] and ethanol-water mixture [136].

According to Table 1, the photothermal effect of GNRs to convert NIR to heat, is the most applied GNRs’ feature in drug delivery or therapeutic systems. Considering the systems’ functional mechanism, this property is mostly used for tissue hyperthermia in photothermal therapies or a local drug release [95,112]. This feature is also effective in increasing the cytotoxicity of drugs such as DOX [107].

Coupling GNRs with materials such as mesoporous silica would keep GNRs from aggregation and enhance the system’s drug loading capacity [137]. In many designed systems, the Au-core provides stability, and the monolayer shell tunes the desired surface features such as charge or hydrophobicity. The disadvantages of GNRs, such as high aggregation rate and short blood circulation lifetime, could also be improved by modifying them with polymeric materials [108]. Additionally, GNRs are promising candidates for theranostic applications, due to their imaging and thermal-induced therapy features. [117].

In general, to come up with the best choices for a gold nanoparticle-based drug delivery system design, investigation of the cell penetration rate of the carrier and the factors affecting it (e.g., surface chemistry, functionalization, geometry, morphology, and size), the assessment of the carrier’s effects on the cargo, and the induced biological responses is highly crucial. The complex behavior of GNRs and GNPs was studied by Pyshnaya et al. The study revealed that cell penetration of both positively charged GNRs and GNPs was better than negatively charged ones. It was also indicated that the initial surface charge was even more important than their final charge in the biological fluids. Therefore, it is assumed that GNRs and GNPs have almost similar cell uptake rates (natural and cancerous cells) based on their functionalization [93]. However, the surface charge does not seem to be the only effective factor. GNRs and GNPs were also compared for penetration in antigen-presenting cells (APCs) and humoral response in a study by Niikura et al. Despite the higher antibody titer against GNPs, GNRs had more cell uptake due to the higher cell-entrance to cell-exit ratio. In addition, other evidence such as different cytokine measurements indicated that GNRs had the lysosomal escaping ability and activated the inflammasome pathways, while GNPs readily exit the cell after the entrance. In other words, the morphology of gold nanostructures (GNPs/GNRs) could affect immune responses via activating nano inflammasome dependent manner [138].

### 3.2. GNRs for Gene Delivery Systems

**Figure 2:**
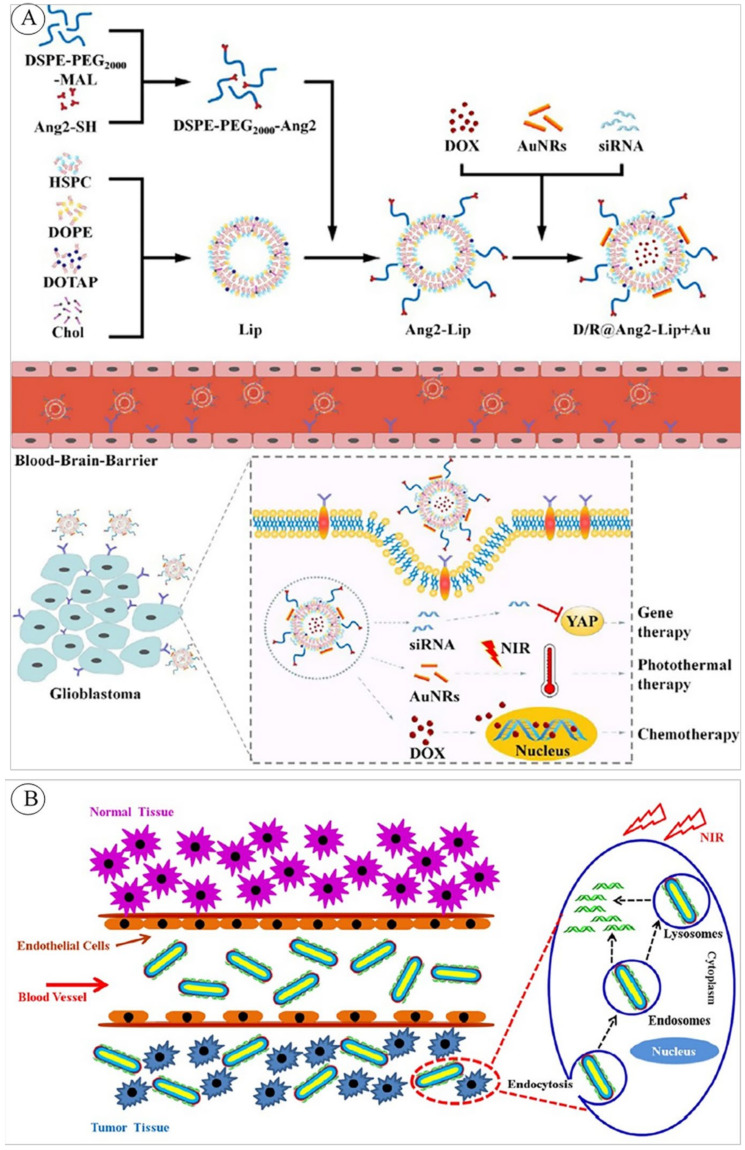
(**A**) The designed nanosystem for a combination of chemo, thermal and gene delivery for glioblastoma. Reprinted from [131] with permission from Elsevier, Amsterdam, Netherlands. (**B**) Chitosan-GNR-based delivery system for siRNA-mediated cancer therapy. Reprinted from [140] with permission from Elsevier, Amsterdam, Netherlands.

GNRs could also be applied for targeted oligonucleotide delivery [139]. For example, DNA and RNA strands are stabilized on the surface of GNRs in different nanosystem designs for brain drug delivery. GNRs have been applied in a nanosystem designed for glioblastoma therapy by combining thermal therapy and inhibiting a protein expression, which is expected to make the glioblastoma more sensitive to chemotherapy [131]. The system consists of a liposome with a cationic-charged surface, modified by angiopep-2 peptide and loaded internally with DOX (a chemotherapeutic agent) and externally with GNRs (photothermal therapeutic agents) and yes-associated protein siRNA (YAP-siRNA), responsible for inhibiting the expression of YAP protein (Figure 2A). The system showed good tumor growth inhibition effects and could pass the blood–brain barrier [131].

GNRs have also been found useful in successful small interfering RNA (siRNA) delivery in cancer treatments. The application of siRNAs as important cancer treatment agents due to the ability to suppress protein expressions is challenging, since they have poor cellular uptake and rapidly degrade when exposed to RNase and serums or endosomes inside the cells. A nano siRNA-delivery system has been designed by assembling chitosan on GNRs (Figure 2B). This system had sufficient cargo protection, successful cell and tissue delivery and good endosomal escape, thus effective in cancer suppression. The photothermal property of GNRs was also helpful in enhancing the treatment efficacy [140]. Having a remarkable photothermal property, GNRs are now considered promising candidates for designing controlled-release systems that can be activated by near-infrared radiation. Shuji Yamashita et al. developed such a system by modifying dsDNA on GNRs. Irradiating GNRs by NIR resulted in the release of ssDNA due to the photothermal effect, during which the amount of release could be controlled by the power and time of radiation [141].

Combinational systems for gene delivery, PTT and thermal imaging are also achievable using GNR. In a recent study GNRs were grafted by poly(amidoamine) dendrimers and modified by GX1 peptide to design a system for simultaneous gene delivery (FAM172A gene), photothermal therapy and thermal imaging for tumor treatment [142]. A summary of the research in gene delivery applications of GNRs is provided in Table 2.

According to the above table (Table 2), the most important and the only reason for using GNRs in gene delivery applications is their photothermal effect. In addition, siRNAs and shRNAs are the most used nucleic acid-based cargoes in GNRs delivery systems. This may be due to the silencing ability of these cargoes for disease treatment, which has fewer unknown side effects than permanent DNA therapies [143]. One of the widely used nanoparticles with similar morphology to GNRs, is carbon nanotubes (CNTs). GNRs and CNTs are both one-dimensional nanoparticles with a high ability to bind to nucleic acids. They have high surface-to-volume ratios, ease of functionalization, and optical and conductive abilities. Additionally, they both represent a good photothermal effect. Their differences are that CNTs are hollow cylinder-shaped carbon-based nanoparticles while GNRs are filled and solid cylinder-shaped metal nanoparticles. Nucleic acids are mostly absorbed physically and electrostatically to the CNTs, while in GNRs delivery systems, covalently bonded nucleic acids are used with the same amounts as the electrostatically bonded ones. One important issue that limits the clinical use of CNTs in comparison with GNRs is their nanotoxicity or environmental effects [144]. Fabricating hybrid structures based on GNRs and CNTs could be an intriguing and novel delivery platform in future gene delivery systems [145].

**Table 2:** GNRs applications in gene delivery systems (research-based studies).

Purpose	System’s Structure	Mechanism of Function	GNR’s Applied Feature	Ref.
RnD	Dendrimer coated GNRs	Delivery and enhanced expression of the brcaa1-shRNA to the targeted cells upon NIR irradiation	PT	[146]
DnD	Disulfide cross-linked polyethyleneimine-conjugated GNRs grafted by PEG and RGD peptide	Gene release in response to high glutathione concentration in target cells and NIR irradiation	PT	[147]
RnD	Layer-by-layer assembled chitosan-GNRs	Delivery of siRNAs, accumulation in tumor tissue, NIR-mediated photothermal ablation	PT	[140]
DnD	Cationic-charged surfactant and DNA modified GNRs	DNA release as a result of Photothermal hyperthermia	PT	[148]
Drug +siRNA co-delivery	DOX, YAP-siRNA and GNRs loaded cationic liposome	Targeted chemo, PT and gene combination therapy using NIR irradiation	PT	[131]
DnD	GNRs grafted with Poly(amidoamine) dendrimers and modified by GX1 peptide, FAM172A gene	DNA release and PTT as a result of Photothermal hyperthermia	PT	[142]

Abbreviations: DnD: DNA delivery, RnD: RNA delivery.

### 3.3. GNRs for Photothermal/Photodynamic Therapy

Therapeutic applications of GNRs are mainly based on their photothermal effect. The photothermal effect of GNRs can deteriorate tumors and cancerous cells using local irradiated heating, called photothermal therapy (PTT). Moreover, it can occur due to the singlet oxygen generation, called photodynamic therapy (PDT) [11,72]. In the last few decades, numerous studies have been carried out on this topic to improve fabrication and assemble optimal photothermal systems. Conventional methods of hyperthermia affect both tumors and healthy cells, so they have lower specificity. In addition, a temperature gradient forms on the way to the tissues, which provides a more severe issue for deep tumors. Nanoparticle-based hyperthermia can facilitate these limitations by targeting the tumor tissue and then generating local hyperthermia. Among the most essential nanostructures capable of generating hyperthermia, such as gold nanoparticles, magnetic nanoparticles (MNPs), CNTs, and ZnCuO [149], gold nanorods have remarkable capabilities to make them ideal for thermal therapy [150].

A comparison of gold nanorods with gold spherical nanoparticles shows that although gold spherical nanoparticles (GNPs) have the photothermal effect, they have several limitations. Indeed, GNPs not only have relatively low heating efficiency, but also the wavelength at which their resonance occurs, located in the mid-visible region that is outside of the “tissue window”. Therefore, the use of gold nanorods is a significant advantage because they are more efficient photo converters to heat and can coordinate the maximum heat wavelength within the tissue window [151]. However, several studies have been conducted on the PTT applications by means of GNPs [152,153].

In this case, MNPs have also the great potential of heat generation. However, the effectiveness of MNP hyperthermia treatment greatly depends on some magnetic parameters, such as alternating magnetic field (AMF), intensity of the AMF, penetration depth. For example, exposure to a high frequency alternating magnetic field is needed to convert electromagnetic energy into heat energy. Moreover, bio functionalization in addition to the targeted delivery, plays an essential role in generating the thermal stability of the particles. The targeted heating of MNPs is lower than GNRs, due to the fact that biomolecules are often physically bonded to MNPs. In contrast, GNRs could covalently and electrostatically be functionalized with a variety of biomolecules. Thus, bio functionalization is mostly less stable, and the heating damage of healthy tissue or overheating is possible. Recent studies in this area have focused on proposing next generations of MNPs such as superparamagnetic iron oxide nanoparticles (SPIONs), due to their unique optical and magnetic properties that help to decrease the overheating risks and enhance the heating efficacy. In addition, according to the highlighted optical properties of CNTs, they also express great potential of tumor ablation in both targeted and non-targeted manner. Despite the advantages of CNTs, the safety aspects of using them in vivo, including toxicity and different antitumor mechanisms of action, are still under investigation [154].

**Figure 3:**
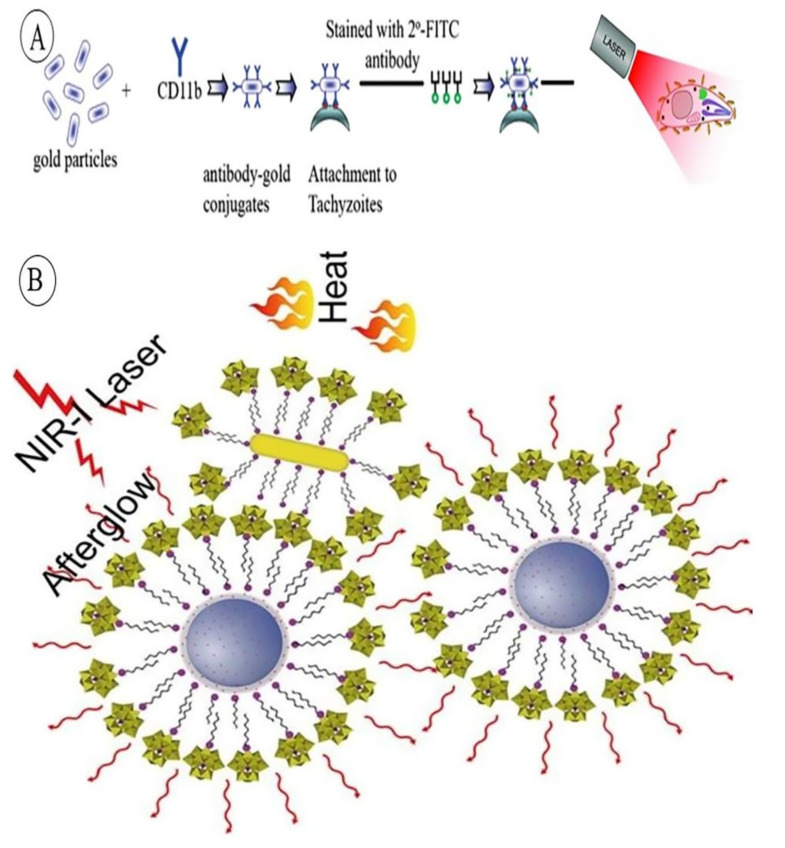
(**A**) Schematic figure of GNRs attachment to targeted tachyzoit cells. Reprinted from [155] with permission from ACS Publications, Washington, DC, USA. (**B**) Scheme of PLNP-GNRs biocompatible nanocomposite platform for NIR-induced photothermal tumor therapy. Reprinted from [158] with permission from Elsevier, Amsterdam, Netherlands.

Several studies in the last two decades showed that photothermal therapy can be implemented for different sort of cells as well. In this case, Pissuan et al. could empirically assemble the system using functionalized GNRs with anti-CD11b (Tachyzoite surface antigens) to targeted delivery of a nanosystem. Thus, increasing the temperature showed promising results in killing the active protozoans, hampering the parasite life cycle (Figure 3A) [155].

In another study, Lee et al. have designed a new kind of virus-simulated system, using necotin receptor peptides (RVG) as coverage of PEGylated silica-coated gold nanorods (SiO_2_@GNRs-PEG) nano assembly, not only to transport the system through the blood–brain barrier but also to inhibit the growth of glioma tumor using hyperthermia. Results showed that implementing the system could lead to locally increasing the temperature above 50 centigrade just after 5 min of NIR exposure [156].

It is noteworthy that despite the number of studies focusing on GNR-mediated PTT, the research has not yet reached the end. In the most recent study in 2021, on hospitalized coronavirus patients, scientists tried to use a more external and less invasive medication procedure for patients with severe respiratory complications. So, functionalized GNRs with angiotensin-converting enzyme 2 (ACE2) have been chosen at the first line of treatment to degenerate targeted cells, inducing apoptosis or necrosis [157]. Additionally, in another study, by Meng et al., persistent luminescent nanoparticles (PLNPs)-conjugated GNRs have been used, forming a nanocomposite platform (PLNP-GNRs) which were encapsulated by biocompatible PW_12_ molecules (phosphotungstate derivatives). The results revealed that GNRs can effectively act as a promising photothermal conversion agent, using single 635 nm wavelength, for targeted photothermal therapy of deep-tissue tumors, particularly in skin and breast cancer cases (Figure 3B) [158].

Table 3 summarizes some of the photothermal therapeutic systems in which GNRs show photothermal effects.

## 4. GNRs for Theranostics; Combination of Diagnostic and Therapy

**Figure 4:**
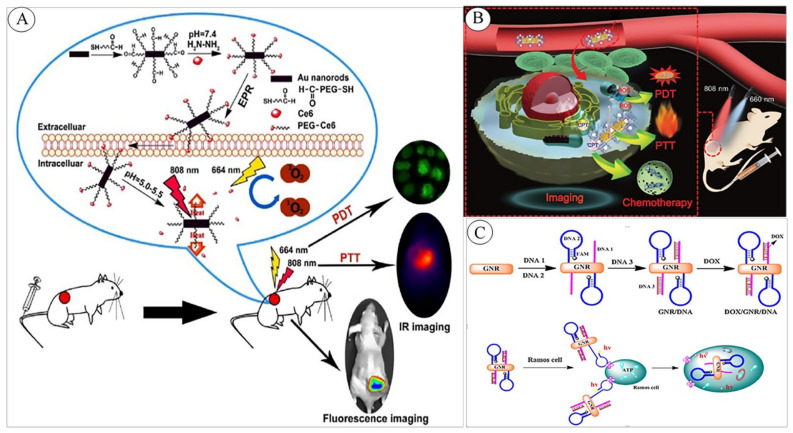
(**A**) Schematic figure of the theranostics designed nanosystem, using pH-induced chlorin e6@ gold nanorods (Ce6-PEG-AuNR). Reprinted from [72] with permission from Elsevier, Amsterdam, Netherlands. (**B**) Schematic illustration of AuNR@MOFs@CPT theranostics nanosystem for tumor’s multiplexed photodynamic/photothermal/chemotherapy. MOF represents as metal-organic frameworks. Reprinted from [172] with permission from Wiley Online Library. (**C**) a dual functional GNR-based system for targeted drug delivery and intracellular ATP detection. Reprinted from [173] with permission from Elsevier, Amsterdam, Netherlands.

The integration of diagnostic imaging and therapy, called theranostics, has emerged as an advancing field for high-performance cancer treatment. Hence, the majority of current studies have been reported on developing potential cancer theranostic agents. According to the imaging techniques, bio-imaging is universally preferred, as it is a non-invasive procedure for in situ visualization and offers real-time tracking of biological processes. So, GNRs are an excellent candidate for bio-imaging and photothermal therapy, due to the capability of local heat generation, triggered by laser beam [165]. GNRs can be applied solely or simultaneously by other imaging techniques such as PA imaging and positron emission tomography (PET) to improve both the imaging’s resolution and image-guided therapy [166,167,168,169,170,171]. Herein, Zhang and his colleagues designed a nanosystem as a theranostic platform by using chlorin e6 (fluorescent tag)-conjugated gold nanorods (Ce6-PEG-GNRs), resulted in enhancing the efficiency of therapy, because of dual PTT/PDT, and precisely-tumor tracking of nanosystem by pH-responsive fluorescent and IR imaging (Figure 4A) [72]. The combination of multimodal therapeutic method (photothermal, photodynamic and chemotherapy) and diagnosis was also shown in another study in which two different wavelength laser radiation (660 nm, 808 nm) were used to elevate the effect of treatment (Figure 4B) [172]. Drug delivery potency of GNRs can also be integrated with biomarkers to track a drug’s local release. Guo et al. designed such a system for targeted delivery of DOX and detection of intracellular ATP. The system was composed of GNRs, modified by three types of DNA (DNA1: complementary DNA sequence for ATP aptamer, DNA2: Ramos cell aptamer, DNA3: ATP aptamer) and loaded by DOX. The immobilization of ATP aptamers and Ramos cell aptamers made GNRs capable of specific recognition of tumor sites. The presence of ATP caused the release of DOX from the nDDS and increased its fluorescence intensity related to the ATP concentration (Figure 4C) [173].

The application of gold nanorods in some of the theranostic systems are mentioned in Table 4.

## 5. Conclusions

This study focused on the properties, synthesis, and applications of gold nanorods (GNRs), as promising candidates for utilization in nano-drug delivery systems (nDDS), photothermal therapy, rapid diagnostic and imaging, and theranostics applications. Among these applications, the capacity of GNRs in converting irradiation into heat has been considered the most distinctive feature in photothermal therapeutic strategies and localized drug release. This feature is also shown to be effective in drug diffusion and increase the cytotoxicity of drugs such as doxorubicin. Moreover, studies have shown the possibility of using an efficient hybrid system of GNRs with a wide range of materials such as SiO_2_, nucleic acids, polyvinyl alcohol, chitosan, magnetic ionic liquids, hyaluronic acid, graphene oxide, polymers, peptides and potential drugs.

Nevertheless, the interaction between GNRs and different chemical/biological elements and the possibility of unfavorable, irreversible changes in their physicochemical characteristics is an important issue that needs deep research and ex/in vivo experiments for nDDS and other potential applications, i.e., nanobiosensing and regenerative medicine. Manageable surface chemistry, high stability (less agglomeration and corrosion), low cytotoxicity and high biocompatibility, simple synthesis protocols with reproducibility in scaling up, strong signals for bioimaging, sustainability in delivery, and cost-effectiveness are also other concerns that need to be addressed in designing such hybrid systems.

Today, nDDS has shown to be a promising strategy for diverse applications. However, there are serious concerns in a biological medium such as immune response elicitation, the toxicity of nanoparticles, enhancement of the capacity and efficiency of drug loadings, unpredictable degradation patterns of nanoparticles inside the body, and possible environmental contaminations. Therefore, apart from optimizing synthesis protocols and stabilizing functional nanocarrier systems, understanding their biological effects/interactions in the host system would be important.

The anisotropic morphology of GNRs provides them a good potential for biomedical usage. Besides their applications in the field of drug delivery, developing new electrochemical and optical sensors are promising areas in the future of GNRs. Photothermal and photodynamic therapy based on the GNRs are also important especially due to their usage in the tumor ablation applications. Furthermore, current positive results in clinical trials based on plasmonic photothermal therapy of atherosclerosis with GNPs show that market of GNRs will be a bright one in the biomedical field and overtake the rivals, because of the highlighted GNRs privileges among other plasmonic nanoparticles such as silver or even gold nanoparticles. Despite the presence of two drug formulations based on the gold nanospheres, currently in clinical trial processes, there are no FDA-approved therapeutic formulations based on the GNR’s delivery system in the clinical trials until 2021. Aurolase is a core–shell silica gold nanoparticle (GNPs) coated with PEG and used for tumor ablation based on the photothermal effect of AuNPs (GNPs). Lung, head and neck, and prostate cancers are the diseases being investigated to treat solid tumors with this drug. NU-0129 is another drug consisting of spherical gold nanoparticles (GNPs) functionalized with nucleic acids, which targets the Bcl2L12 gene. This gene hampers the apoptosis in the glioblastoma cells, so blocking this gene through NU-0129 is a treatment for patients with recurrent glioblastoma or gliosarcoma. These cases show that GNRs could have good potential for future applications in gene delivery and photothermal applications.

## Figures and Tables

**Table 1:** GNR applications in drug delivery or therapeutic systems (research-based studies).

Purpose	System’s Structure	Mechanism of Function	GNR’s Applied Feature	Ref.
DD	Folate targeted GNRs, cDNA and tether platinum [Pt(IV)]	Photothermal hyperthermia and dual drug release with the NIR-laser stimulation	PT	[100]
DD	NIR-responsive chromophore, b-cyclodextrins functionalized PEG, DOX-loaded GNRs	NIR-stimulated drug release	PT	[101]
DD	Biotin targeted DNA-conjugated GNRs loaded by DOX	NIR and pH-induced drug release	PT	[95]
DD	GNR core, magnetic ionic liquid, and DNA moieties shell	Photothermal hyperthermia, drug release	PT	[102]
DD, PTT	Hyaluronic acid and RGD (Arg-Gly-Asp)-conjugated silica-coated GNRs, loaded by DOX	Photothermal hyperthermia, pH-enzyme sensitivity, and NIR-triggered drug release	PT	[103]
PD	GNRs coated polysaccharide-based hydrogel loaded by fluorescein isothiocyanate-modified ovalbumin (FITC-OVA)	Photothermal hyperthermia and enhanced transdermal FITC-OVA delivery	PT	[96]
DD, PTT	Graphene oxide-conjugated GNRs loaded by DOX	Photothermal hyperthermia and NIR-stimulated drug release	PT	[104]
DD, PTT	GNRs with glutathione responsive diblock co-polymer micelles loaded by GW627368X	high glutathione concentration-responsive drug release, PTT-induced apoptosis, and necroptosis	PT	[105]
DD	GNR core, mesoporous silica/hydroxyapatite shell	NIR- and pH-induced drug release	PT	[106]
DD, PTT	DOX-loaded GNRs with a pH-sensitive linkage	pH responsive DOX release, Photothermal hyperthermia	PT	[107]
DD, PTT, PDT, Im	Hyaluronic acid (HA) functionalized GNRs, conjugating with 5-aminolevulinic acid (ALA), Cy7.5 and anti-HER2 antibody	HER2 and CD44 targeted system. pH, glutathione, and hyaluronidase-responsive drug release. NIR-stimulated PDT and PTT.	PT	[97]
DD	Stimuli-responsive ABC triblock co-polymer coated GNRs loaded by DOX	DOX release in response to pH and thermosensitive ABC triblock co-polymer	St	[108]
DD, PTT	Conjugate of dihydroxy phenyl/hydrazide bifunctionalized hydroxyethyl chitosan-GNR, decorated with a pH-sensitive oxidized hyaluronic acid and loaded by DOX	CD44 targeted, pH-responsive drug release, PTT	PT	[109]
DD, Im	Au@Ag core–shell nanorod coated by zeolitic imidazolate framework (ZIF-8) with 4-aminothiophenol (4-ATP) as reporter molecule, loaded by DOX	Cell entrance through endocytosis, Enhanced drug’s therapeutic effects	Ca	[110]
DD	DOX loaded Chlorella, GNRs and BSA-based Gel	Irradiation of 660 nm and 808 nm for oxygen and heat generation by Chlorella and GNR, respectively	PT	[111]
DD	GNRs incorporated with Poly (*N*-isopropylacrylamide)-*co*-acrylamide polymer, loaded with a model drug	Photothermal hyperthermia and local drug release	PT	[112]
DD	GNR core, mesoporous silica shell, functionalized with *d*-α tocopherol PEG 1000 succinate and PEI, loaded by DOX	NIR-activated drug release	PT	[113]
DD	PEGylated graphene oxide capped-GNR/SiO_2_ core–shell, loaded by DOX	Dual NIR- and Ph-activated drug release	PT	[114]
DD, GD	C(HK)4-mini PEG-RGD peptide-functionalized GNRs	αvβ3 integrin targeted, Cell uptake by RGD (Arg-Gly-Asp) peptide, HK (His-Lys) peptide-conjugated delivery	Ca	[115]
DD, PTT	cRGD peptide-modified GNRs loaded by paclitaxel and curcumin	αvβ3 integrin targeted by cRGD, NIR-responsive drug release and tumor growth inhibition by apoptosis	PT	[116]
DD, PTT	GNR @ silica coated/poly histidine/*d*-α-tocopherol PEG succinate/DOX-loaded PEGylated nanocomposite	NIR thermal-/pH-triggered drug release and PTT	PT	[117]
DD, PTT	GNRs coated by folate-α,β-poly(*N*-2 hydroxyethyl)-d,l-aspartamide, loaded by Antineoplastic drug irinotecan (Iri)	Folate-targeted drug release, Photothermal hyperthermia	PT	[118]
DD, PTT	GNRs@Kr (Keratine), loaded by DOX	NIR-triggered and redox-responsive drug release	PT	[119]
DD, PTT	Hollow H-apatite capped (poly(*N*-isopropylacrylamide-co-acrylic acid) GNRs-DOX loaded hybrids microcapsules	pH/NIR-responsive DOX release	PT	[120]
DD	GNR-polydopamine capped hollow h-apatite hybrid microcapsules	pH/NIR-responsive drug release	PT	[121]
DD	GNR-chitosan modified by poly(l-lactide), poly(*N*-isopropylacrylamide) and poly (acrylamide) micelles, loaded by paclitaxel	light triggered DD and PTT	PT	[122]
DD	Alginate/polyacrylamide hydrogel patch -hybridized by polyvinylpyrrolidone- graphene oxide@GNRs	NIR-stimulated thermoresponsive methotrexate and rhodamine B co-delivery	PT	[123]
DD	Multi-layer allylamine hydrochloride/poly methacrylic acid capsules –DOX/nimbin co-delivery	NIR-stimulated drugs release	PT	[124]
DD, PTT	Folate-modified liposomes encapsulating NF-κB decoy oligodeoxynucleotides, GNRs, and dexamethasone	Therapeutic effects by anti-inflammatory agents delivery and PTT	PT	[125]
DD, Th	Core–shell mesoporous silica-coated GNRs, deposited by the metal-organic framework, modified by hyaluronic acid, loaded by DOX	Laser irradiation-mediated imaging and drug release	PA/PT	[126]
DD, Im	GNRs coated by poly platinum (polyPt(IV))	Photothermal hyperthermia, redox-triggered polyPt(IV) release	PA/PT	[127]
DD, PTT	PLGA-PEG polymeric corona loaded by vincristine encapsulating silica-coated GNRs	pH-responsive sustained drug release and PTT	PT	[128]
DD, PTT, PDT	Hyaluronic acid encapsulated, GNRs coated with mesoporous and degradable silica, loaded with DOX and IR820 (a photosensitizer)	Hyaluronidase and high glutathione concentration-targeted, triple therapy by NIR irradiation (PDT, PTT and chemotherapy)	PT	[129]
DD	GNRs decorated Polymethacrylate co-polymers and ciprofloxacin	Targeted delivery to bacteria and biofilms, PT effect and antibiotic delivery	PT	[130]
DD, GD	DOX, YAP-siRNA and GNRs loaded cationic liposome	Targeted chemo, PT and gene combination therapy using NIR irradiation	PT	[131]
DD	GNRs-doped hydrogel, made of hyaluronic acid, PEG diacrylate/4-vinyl phenylboronic acid and Astragaloside IV nanoparticles	Sustained drug release, as conductive as native myocardium	Co	[99]
DD, PTT	DOX, Mesoporous silica-capped GNRs covered with selenium nanoparticles shell (Se@Au@mSiO2)	Chemo-drug, selenium nanoparticles as anticancer agent, and PTT ability of GNRs as cancer therapy system	PT	[132]

Abbreviations: PT: photothermal, DD: drug delivery, PD: protein delivery, PTT: photothermal therapy, PA: photoacoustic, Co: conductivity, Ca: carrier, St: stability, PDT: photodynamic therapy, Im: imaging, GD: gene delivery, Th: theranostic, siRNA: small interfering RNA, YAP: yes-associated protein, PLGA: poly(lactic-*co*-glycolic acid).

**Table 3:** GNR-based photothermal (PT) effects in photothermal therapeutic systems (research-based studies).

Purpose	System’s Structure	Mechanism of Function	Ref.
TD, PTT	CD11b antibody @ GNRs	NIR-induced targeted protozoan cell (tachyzoites) death	[155]
PTT	BSA-coated GNRs-laden-macrophages	RAW 264.7 macrophages, hepatic satellite cells (HSCs), HepG2 photo-induced cell damage, leading to lower tumor recurrence	[159]
PTT, PDT	GNRs @ silicon 2,3-naphthalocyanine dihydroxide stabilized with alkylthiol-conjugated polyethylene glycol	PD & PT synergistic therapy, using a single specific laser wavelength	[160]
PTT	Encapsulated GNRs by lipid-based envelope	Good cellular uptake, enhanced NIR-induced PTT, caused cancer cells apoptosis	[161]
TD, PTT	Virus inspired therapeutic system (RVG 29-binded PEG-SiO_2_@ GNRs)	Rabies virus glycoprotein (RVG)-guided for BBB crossing, brain glioma targeted PTT	[156]
TD, Cancer PTT	GNRs combined with a Nestin binding peptide	PT-induced Nestin positive of Glioblastoma Multiform tumors cell apoptosis	[162]
PTT	Silica coated Au-TPGS (vitamin E polyethylene glycol succinate)-HA co-polymer	NIR-induced thermal cancer cell death	[163]
TD, PTT	ACE-2-functionalized GNRs	NIR-induced thermal SARS-CoV-2 cell damage	[157]
Gas synchronous PTT	GNR- polydopamine (PDA) core–shell nanostructure doped by Benzothiazole sulfinate (BTS)	pH triggered BTS (SO2 prodrug) release, resulting in deep tumor gas therapy and PTT	[164]
PTT	PLNP-GNR nanocomposite platforms consisting of ZGGO:Cr^3+^@CTAB@PW12 and GNR@CTAB, PW12- encapsulated	Persistent luminescent nanoparticles combined with GNRs as biocompatible platform for photo-induced therapy.	[158]

Abbreviations: TD: targeted delivery, PT: photothermal, PTT: photothermal therapy, PDT: photodynamic therapy.

**Table 4:** GNR applications in theranostics systems (research-based studies).

Purpose	System’s Structure	Mechanism of Function	GNR’s Applied Feature	Ref.
DD, TRK	GNRs@folic acid as the targeting ligand the anthracycline drug, DOX	Targeted drug delivery to cancerous cells (expressing folate receptor cells), fluorescence lifetime imaging, using photoluminescence of GNRs and the innate fluorescence of DOX	PL	[174]
DD, TRK	Au@mesoporous SiO_2_/rhodamine B isothiocyanate (Au@mSiO_2_/RBITC) nanocomposite	Monitoring the photothermal therapy, drug release, cell tracking using an 808 nm laser and a confocal laser scanning microscopy system	PT	[175]
DD, In situ ATP TRK	DNA-functionalized GNRs loaded DOX	Targeted drug delivery, drug release in response to aptamer–ATP interaction, tumor growth inhibition and increasing fluorescence emission of DOX proportional to ATP concentration	PT	[173]
PTT and Im	PEG-peptide-modified GNRs containing peptide substrate, overexpressed in malignant tumor cells	Formation of GNR aggregates in response to uPA activity and PEG-chains release and absorption reduction in tumor’s site	PT	[176]
PTT, PDT, Im	Ce6-doped mesoporous silica- nanorods (AuNRs-Ce6-MSNRs)	Generation of hyperthermia by GNRs (PTT) and singlet oxygen (1O2) production by Ce6-mediated PDT. Dual-imaging by the photoacoustic and NIR-induced fluorescence of AuNRs & Ce6	PT/PA	[177]
Th	chlorin e6 (Ce6)-PEG-GNR	PTT and PDT, using pH-responsive Ce6 (fluorescent tag)-based local tumor tracking and dual IR imaging	PT/PDT	[72]
PTT, PDT, Im	AuNR@MOFs nanocomposite loaded CPT	photothermal-induced CPT release and photodynamic combinational therapy	PT/PDT/PA	[172]
Th (Im, PDT & PTT synergy/TD	Pheophorbide-responsive glutathione functionalized GNRs, conjugated folic acid-PEG co-polymer	Light triggered imaging and NIR-induced synergistic therapeutic	PT/PDT	[178]
Im, PTT	Graphene oxide (GO)-GNR contrasting thermal conductivity capacity and heating therapy.	NIR-induced imaging and thermal stone dissolution	PA/PT	[179]
Th (Im, PDT, PTT, TRK)	(PNIPAM) coated SiO_2_@GNR loaded ICG (nanocom-ICG), thermal-induced targeted release for tumor therapy	NIR-induced tracking and enhancing the thermal release of ICG-mediated photodynamic therapy	PA/PT	[180]
Th	Eu@SiO_2_@GNR complex as multiplexed PT agent, photo and radio sensitizer	Image-Guided Photothermal and X-ray-induced Photodynamic Therapy of Tumors	PT/PDT/PA	[181]

Abbreviations: TRK: tracking, TD: targeted delivery, PL: photoluminescence, PT: photothermal, PA: photoacoustic, Im: imaging, PTT: photothermal therapy, PDT: photodynamic therapy, Th: theranostics.

## Data Availability

Not applicable.

## Abbreviations

GNR	Gold nanorods
NIR	Near-infrared
LSPR	Localized Surface Plasmon Resonance
nDDSs	nano-Drug delivery systems
BBB	Blood–brain barrier
CTAB	Cetyltrimethylammonium bromide
PE	Polyelectrolytes
PEG	Polyethylene glycol
DIC	Disseminated intravascular coagulopathy
DVT	Deep vein thrombosis
ROS	Reactive oxygen species
GNPs	Gold nanoparticles
PEI	Polyethylene imine
BSA	Bovine serum albumin
EPR	Permeability and retention
IFP	Interstitial fluid pressure
DOX	Doxorubicin
MDR	Multidrug-resistant
ALA	5-Aminolevulinic acid
PT	Photothermal
DD	Drug delivery
PD	protein delivery
PTT	photothermal therapy
PA	Photoacoustic
Co	conductivity
Ca	Carrier
St	Stability
PDT	photodynamic therapy
Im	Imaging
GD	Gene delivery
Th	Theranostic
siRNA	small interfering RNA
YAP	Yes-associated protein
PLGA	Poly(lactic-*co*-glycolic acid)
CNTs	Carbon nanotubes
DnD	DNA delivery
RnD	RNA delivery
MNPs	Magnetic nanoparticles
AMF	Alternating magnetic field
ACE2	Angiotensin-converting enzyme 2
TD	Targeted delivery
PET	Positron emission tomography
TRK	Tracking
PL	Photoluminescence
